# Integrated IoT-Based Secure and Efficient Key Management Framework Using Hashgraphs for Autonomous Vehicles to Ensure Road Safety

**DOI:** 10.3390/s22072529

**Published:** 2022-03-25

**Authors:** Sudan Jha, Nishant Jha, Deepak Prashar, Sultan Ahmad, Bader Alouffi, Abdullah Alharbi

**Affiliations:** 1 School of Sciences, Christ (Deemed to be University), NCR-New Delhi Campus, Ghaziabad 201003, India; jhasudan@ieee.org; 2 Department of Networking and Security, School of Computer Science and Engineering (SCSE), Lovely Professional University, Phagwara 144411, India; nishantjha1998@outlook.com (N.J.); deepak.prashar@lpu.co.in (D.P.); 3 Department of Computer Science, College of Computer Engineering and Sciences, Prince Sattam Bin Abdulaziz University, Alkharj 11942, Saudi Arabia; 4 Department of Computer Science, College of Computers and Information Technology, Taif University, P.O. Box 11099, Taif 21944, Saudi Arabia; balouffi@tu.edu.sa; 5 Department of Information Technology, College of Computers and Information Technology, Taif University, P.O. Box 11099, Taif 21944, Saudi Arabia; amharbi@tu.edu.sa

**Keywords:** autonomous vehicles, blockchain, Ethereum, hashgraph, batch rekeying, logical key hierarchy

## Abstract

Autonomous vehicles offer various advantages to both vehicle owners and automobile companies. However, despite the advantages, there are various risks associated with these vehicles. These vehicles interact with each other by forming a vehicular network, also known as VANET, in a centralized manner. This centralized network is vulnerable to cyber-attacks which can cause data loss, resulting in road accidents. Thus, to prevent the vehicular network from being attacked and to prevent the privacy of the data, key management is used. However, key management alone over a centralized network is not effective in ensuring data integrity in a vehicular network. To resolve this issue, various studies have introduced a blockchain-based approach and enabled key management over a decentralized network. This technique is also found effective in ensuring the privacy of all the stakeholders involved in a vehicular network. Furthermore, a blockchain-based key management system can also help in storing a large amount of data over a distributed network, which can encourage a faster exchange of information between vehicles in a network. However, there are certain limitations of blockchain technology that may affect the efficient working of autonomous vehicles. Most of the existing blockchain-based systems are implemented over Ethereum or Bitcoin. The transaction-processing capability of these blockchains is in the range of 5 to 20 transactions per second, whereas hashgraphs are capable of processing thousands of transactions per second as the data are processed exponentially. Furthermore, a hashgraph prevents the user from altering the order of the transactions being processed, and they do not need high computational powers to operate, which may help in reducing the overall cost of the system. Due to the advantages offered by a hashgraph, an advanced key management framework based on a hashgraph for secure communication between the vehicles is suggested in this paper. The framework is developed using the concept of Leaving of Vehicles based on a Logical Key Hierarchy (LKH) and Batch Rekeying. The system is tested and compared with other closely related systems on the basis of the transaction compilation time and change in traffic rates.

## 1. Introduction

Cyber Physical Systems (CPSs) are believed to improve the quality of life of humans, and Autonomous Vehicles (AVs) are one of the major use cases for CPSs [1]. With recent technological advances and a growing interest in developing connected vehicles, various automobile manufacturers have dedicated a significant portion of their resources to developing these emerging technologies as quickly as possible in a race to be the first to reach the highest degree of autonomy [2]. According to international reports [3], it is believed that up to 90% of road accidents can be prevented by using autonomous vehicles, and it may be one solution to resolve current challenges such as road accidents and congestion control [4]. Recent advancements in disruptive technologies such as blockchain technology have opened new horizons for autonomous vehicles [5]. The successful deployment of AVs is dependent on the creation of a suitable environment that can enable two-way communication between the vehicles and their surroundings. Technologies such as the Internet of Things (IoT) have taken this in the right direction. The use of blockchain technology [6] can upgrade the existing systems, which can revolutionize the future of vehicles. The use of IoT can improve data collection, such as road conditions and road traffic analysis, for effective communication between vehicles. These data are stored centrally, which makes the data vulnerable to various attacks and can reduce the ability of the vehicles to communicate with each other, leading to accidents.

Autonomous vehicles offer various benefits, but at the same time, there are various challenges that need to be resolved. The major challenge that still needs to be resolved is related to the privacy and security of the data exchanged between two or more vehicles in the network, along with their surroundings, and keeping the identities of all the stakeholders of the network private. The current solutions for achieving a trusted message exchange mechanism are based on group key management techniques [7,8]. However, depending only on key management over a centralized network of autonomous vehicles is not effective in preserving the privacy of all the stakeholders of the vehicular network [9]. Thus, the development of a decentralized system can encourage secure and seamless exchanges of data between vehicles, which can be achieved using blockchain technology. If the vehicular network’s security is compromised, then due to fake and unreliable messages sent by the malicious vehicles, important information, such as road conditions, upcoming traffic signals, etc., cannot be exchanged in real-time and blockchain technology can help in resolving the critical challenge related to secure message transfer in a vehicular network [10,11]. Various studies have been conducted to resolve the challenges faced by autonomous vehicles through blockchain technology. For example, in [12], the authors suggested a blockchain-based key management system in a vehicular ad hoc network (VANET) to automate the realization of updating, registering and deleting the public key of the users in the network. This mechanism is based on a key agreement protocol designed to prevent the VANET from attacks, such as collusion attacks and key tampering attacks. Another study [13] focuses on the development of a trust management algorithm based on blockchain technology to ensure the privacy of the VANET and ensure the data security of the vehicles participating in the network. These studies were found effective, but blockchain also has some challenges that limit the maximum efficiency of autonomous vehicles [14,15,16]. Some of these challenges are: (i) Scalability: For complex networks, transaction processing per second slows down, making blockchain inefficient for large-scale networks. (ii) Interoperability: Most of the blockchain’s operation is based on silos, and these do not communicate with other networks and are inefficient in a peer-to-peer message exchange. (iii) Mining complex blockchain networks and providing a secure environment consumes more energy.

There are some studies that suggest the use of 3G/4G/5G technology and Ultra-Reliable Low-Latency networks with a strong focus on effective traffic control [17,18,19] and to reduce the dependence on blockchain technology. In [19], the authors suggested a multiaccess edge computing (MEC)-based architecture for interactions between autonomous vehicles based on baseband units (BBUs). However, this technique is not viable due to the limited land resources and costly development. Therefore, these limitations of the existing techniques will act as the basis for our work. The major goal of our work is focused on improving the quality of system (QoS) in order to enhance the security for the peer-to-peer exchange of messages in autonomous vehicles through hashgraphs. The major contributions of this paper are as follows:

1. We suggest the use of a hashgraph to enhance the security of the system. A hashgraph works as a graph of connected hash values in which every member is responsible for exchanging messages over a network [15]. The peer-to-peer communication between the nodes is encrypted with hash values and prevented with the Asynchronous Byzantine Fault Tolerance algorithm (ABFT) and ensures fair participation of all the nodes in a network for exchanging messages [15,16]. This will also help in reducing the overall complexity of the system.
2. A comparative analysis is also conducted between blockchain and hashgraph technology to validate the efficiency of the suggested framework.

The rest of the paper is structured as follows. Section 2 deals with related works. Section 3 explains the significance of the work. Section 4 deals with the suggested framework. Section 5 deals with the results, and the paper is concluded in Section 6.

## 2. Related Work

Various studies have been conducted for enhancing the security of smart vehicles using blockchain technology. In [20], the authors utilized blockchain for tracing the certificates for each vehicle for message authentication. In [21], the authors suggested a blockchain-based anonymous reputation system for preserving the privacy of the messages in a vehicular network. In [22], the authors have proposed the concept of a proof-of-event consensus based on blockchain in vehicular networks to ensure data integrity. In [23], the authors have tried to resolve the security issues in VANETs by introducing a Tezos-based vehicular blockchain network. In [24,25], the authors have integrated IoT and Blockchain and suggested a secure vehicular network to prevent road accidents. Wang et al. [26] showed a breach of privacy with Waze using a network of “ghost” cars. Because Waze normally transmits your position to other nearby Waze drivers along with your username and how fast you are moving, this attack’s simulated ghost vehicles will mark the location of a real user along their path [26]. By creating a system that enforces privacy, data creators can be confident that attackers’ compromised networks will not be able to trace their roots. Their contribution will return to them in the long run if they want to be users because they will produce more accurate and sustainable data [2,26]. While this paper did not discuss the prospect of creating a cryptocurrency awarded to data creators, this is a possibility that can be applied in addition to the current architecture, and their efforts may result in tokens being used as an incentive to use less congested roads [2]. In [27], the authors suggested a blockchain-based trust management model integrated with a conditional privacy-preserving announcement scheme (BTCPS) to resolve the challenges related to trust and privacy in vehicular networks. In [28], the authors presented a trust management system based on blockchain and a certificate authority registration mechanism in VANETs to enhance the reliability of the VANETs. In [29], the authors have used a permissioned blockchain to secure smart vehicles. This technique uses a challenge-response data exchange mechanism between road side units and vehicles for monitoring the internal state of the vehicles and finding any malicious activity in the network. In [30], the authors suggested a novel blockchain-based batch authentication mechanism to resolve the data integrity issues and congestion control in the internet of vehicles (IoVs) to be deployed in smart cities. In [31,32], the authors developed novel blockchain-based protocols for trust management in IoVs to enhance the reliability of IoVs.

Current studies, such as those cited above, only focus on the development of blockchain-based framework for effective data sharing, traffic management and congestion control. Some studies also integrate the Internet of Things (IoT) with blockchain technology, which helps with collecting and transmitting crucial data, such as road conditions, weather and road accidents, between the vehicles securely. These techniques are efficient but increase the overall cost of the system when implementing them on a large scale or in low-income countries. Furthermore, using blockchain technology can provide data security, but this is not enough for effective communication between autonomous vehicles. Other factors such as transaction speed and consensus mechanisms must also be considered to extract the full potential of autonomous vehicles. This is the motivation behind using a hashgraph for our work. Hashgraphs offer benefits such as a high transaction speed (greater than a thousand transactions per second) than blockchain (a few hundred transactions per second on popular blockchain platforms, such as Ethereum and Bitcoin), which can increase the robustness of the vehicles. Additionally, a hashgraph is free from ledger-based systems such as blockchain, so it is capable of delivering high performance with low-cost infrastructure and low computation power. Thus, using a hashgraph will help in reducing the overall complexity of the vehicular system.

## 3. Motivation of Work

VANETs have been explored on a large scale to improve road safety. However, due to recent advancements in technology, traditional VANETs suffer from challenges such as the privacy of stakeholders participating in the network and ensuring data integrity. Furthermore, traditional VANETs are vulnerable to various cyber-attacks that can result in network malfunctions, causing road accidents and loss of lives [33,34]. These cyber-attacks and their effects on the system are summarized in Table 1.

**Figure 1:**
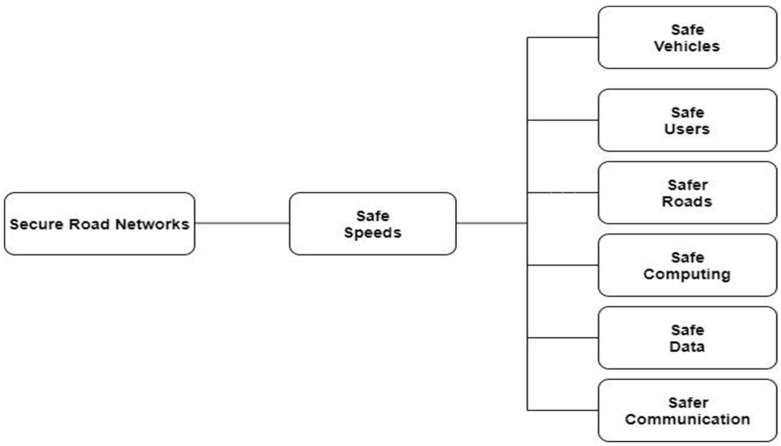
Illustration of a safe system of Interconnected Vehicles [33].

To resolve these threats and keep the vehicular network free from attackers, key management techniques, as discussed in the above sections, are introduced. However, key management alone over a centralized network is not effective for ensuring the safety of the system, as explained in Figure 1. In recent years, researchers have tried to enhance the security of networks using blockchain technology. The idea of blockchain technology is to provide a distributed ledger for digital transactions. These transactions work in a decentralized network of nodes, and only the authentic transactions are allowed to be written on the ledger in the form of blocks. Any change in the block is tracked immediately, and the transaction is declared invalid. This helps avoid transactions and keeps a network safe.

Using blockchain in key management enables the vehicles in a network to communicate rapidly. Each vehicle in a blockchain network will represent a node, and this node has simultaneous and accurate access to all of the information exchanged in the network. This will improve the processing of the transactions occurring between the other vehicles and the roadside units and can help in reducing traffic congestion. This technique has enhanced the security of the VANET but increased the processing time due to the complex structure of blockchain-integrated VANETs. We need to make the system as simple as possible with the fastest information exchange in VANETs. For this purpose, we have introduced a hashgraph-based approach for key management for effective communication between the vehicles.

Hashgraph is a data structure that uses the collaborative concept of “who gossiped to whom” in a particular order. Using hashgraphs for smart vehicles will allow fast and efficient processing of transactions and can enhance the interoperability of the system by securely storing the information in a network. Due to the inherent properties of hashgraphs, the stored information can be verified and validated on the go. Furthermore, the vehicle’s dependency on computation has to take care of Denial-of-Service (DoS) attacks, which affect the computation power of the vehicles in a vehicular network. Using a hashgraph can make the path of communication between the vehicles and other road entities more secure and immutable. The mechanism preferred by hashgraphs is based on the Asynchronous Byzantine Fault-Tolerant (ABFT) mechanism, and it will only permit the hash values of the information to be seen in a network, thereby protecting the information through the nodes and helping in safer communication. In the distributed ledger, the DoS attack affects the nodes in the network and stops the network from working. If the attacker attacks the current node and switches to a new node as soon as one is chosen, then the attacker can freeze the whole system while attacking only one node at a time. This issue is not there in the case of a hashgraph, as no node is provided with special rights to be able to interact with the network; thus, this prevents the chances of DoS attacks. Additionally, road entities engaging in broadcasting with the verification of transactions in a hashgraph would engulf the communication capabilities of the system.

## 4. Suggested Framework

Connected vehicles, beyond traditional autonomous driving, will play a pivotal role in many scenarios. Because of their On-Board Units (OBUs) and data processing capacities, they will grow to work with various stakeholders through their vehicle-to-everything (V2X) connections, both in the physical vicinity and cyberspace. For real-world usage, integrity and privacy are the main issues highlighted. This work presents a hashgraph-based approach to collecting and consuming data about road traffic in the form of an open-source Intelligent Transportation (IT) artifact. Vehicle privacy is assured by using changeable addresses at each interaction with road beacons, which is largely inherited from the security properties of the underlying hashgraph technology, the security of the software on which the application is based [2].

**Figure 2:**
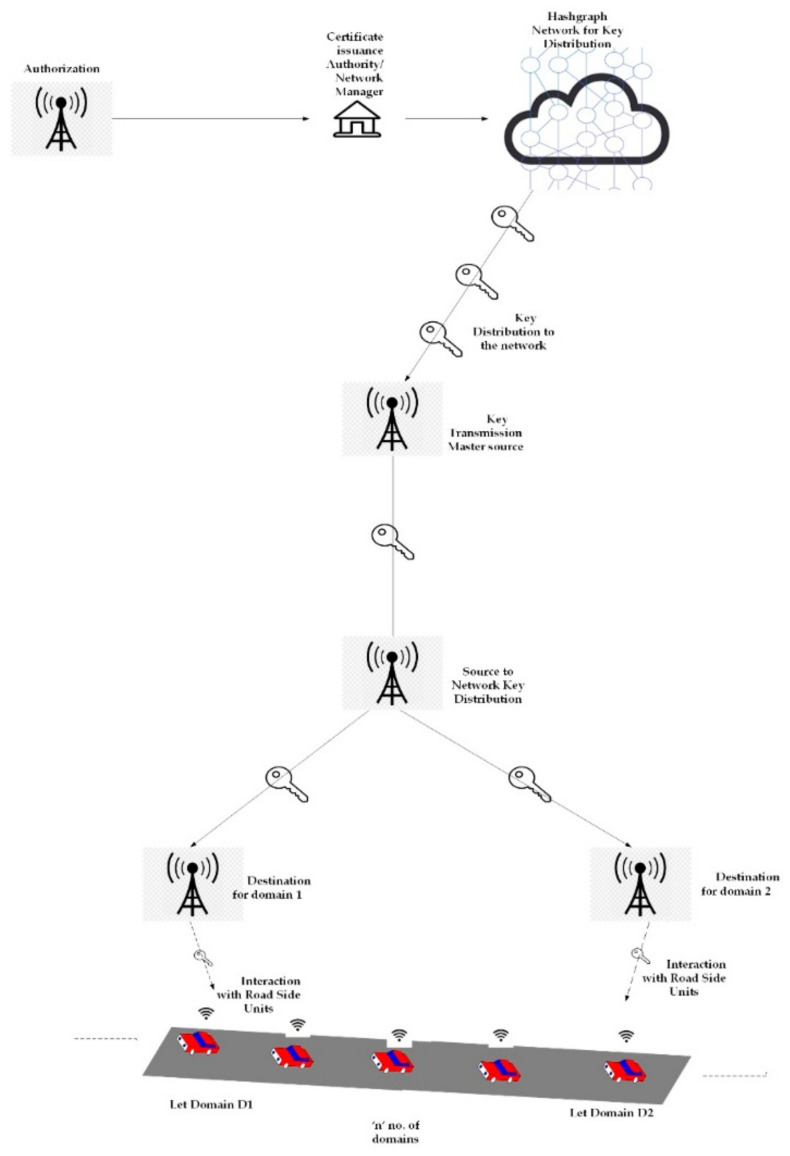
Suggested framework with improvements.

Nowadays, due to their efficient working capacity, most of the autonomous vehicular networks are based on the framework suggested by the authors in [1,4]. The framework suggested by [1,4] is capable of ensuring data integrity of the data exchanged in the network using blockchain technology. The framework’s operation is based on group key management and key handover mechanisms, which control the keys within the network, and the key handover mechanism ensures the transfer of the security keys from one network domain to another to establish an attacker-free network of vehicles. However, due to the complex structure of the blockchain, the efficiency of exchanging messages in the vehicular network will decrease if the network is implemented on a larger scale. Thus, there is a strong need to improve the system’s efficiency, so we have suggested a modified framework based on hashgraphs to enhance the efficiency and security of the vehicular networks. The modified framework is explained in Figure 2.

During vehicular communication, the security of the network can be explained through a group broadcast. Thus, the methodology suggested in this paper utilizes key management, which is considered a better technique than traditional methods to increase the security of the vehicles in a network, thereby improving the communication between the vehicles and hence improving traffic maintenance.

In the suggested methodology, a service authenticator takes over the network manager (different from conventional methods). The centralized service manager is placed separately to generate the cryptographic identities, such as identities of vehicles, and pseudonym certificates for the vehicles that must be kept in a different facility to deal with the security and privacy issues [1,4]. The centralized service managers are verified under the following conditions:

(1) It is necessary for the new vehicles to get registered when they leave from the manufacturing to take part in a new security domain;
(2) All the vehicles must change their pseudonym cryptographic material. They should contact the centralized service manager to obtain a new set of cryptographic materials;
(3) If malicious activities are recognized in a blockchain network, then the identities of adversaries are made publicly available upon the confirmation of those malicious activities.

The hashgraph makes the nodes share information without the need for a centralized institution by enabling a peer-to-peer transaction. The certificate authority has the function of authenticating and connecting other nodes under different domains of security. This network works as a cloud network using a peer-to-peer network. The information in safety instructions is contained in a transaction whenever a handover is requested. This security key is encrypted into a transaction using the public key of its neighboring service managers. This transaction is then shared within the network for authentication purposes. The major function of the handover is the transportation of keys by mining blocks. This helps in maintaining heterogeneous key management in a network. This process is within the local domain of the service manager, and in this way, the keys of the freshly joining members are delivered by extracting the information from the nodes [1,4].

### Probability of Leaving and Leaving Ratio

The probability of leaving a mobile node is defined as the average of the nodes that are leaving the group within a rekeying interval [1,4]. This is explained by Algorithm 1 and Algorithm 2 [1,4].
**Algorithm 1:** Calculation of Probability of Leaving (POL)**Input:** Normal Speed X_C_, Distance Y, maximum positive acceleration a^+^, maximum negative acceleration a^−^, interval between batches T_1_, maximum speed X_lim_ **Output:** POL 1. Maximum speed in $T_{1}\to Z=X_{C}+a.T_{1}$ 2. **if**
$Z\geq X_{lim}$ **then** 3.   keep improving the speed of the vehicles till $X_{lim}$ 4. **else** 5.   keep improving the speed till Z 6. **else if** 7.   $Y_{max}\geq Y$ **then** 8. $S=min\left(Z,X_{lim}\right)$ **else** 9. Set the POL for the node as L=0 10. Minimum speed in $T_{1}\to Z=X_{C}-a.T_{1}$ 11. **if**
$X_{C}.T_{1}\geq Y$ 12.   use Equation (2) to calculate X 13. **else** 14.   use Equation (3) to calculate S 15. **else if** 16.   calculation of max and min speed of the vehicles 17.   calculation of POL is conducted by Equations (2) and (3) 18. **End**

(1) $$\;\;P=\frac{AmountofTraffic}{3600\;s/\mathrm{hour}}*Q*R$$

**Algorithm 2:** Transaction Optimization**Input:** traffic on each road H, m defines transaction optimization timing ($t_{m}^{1}\ldots \ldots \ldots .t_{m}^{n}$) **Output:** optimized transaction timing, $t_{m}$  1. data sink optimization, m  2. **for** (j=1; j≤m; j++) **do**  3. call Equation (4),  4. record P into the result sink  5. return $t_{m}$  6. **End**

The main idea of using this algorithm is to avoid congestion in traffic that could result in road accidents. The entry and exit of nodes are unpredictable in traditional methods, such as in wireless sensor networks and Long-Term Evolution (LTE) networks. Therefore, some key organizational schemes allow nodes that subscribe to multiple rekeying intervals to measure the likelihood of leaving. Sadly, as the program allows users to choose their own subscription period, security flaws appear: a malicious user eavesdrops vital messages by requesting for an active period longer than the real residence duration. Probability-based models are much more effective than the conventional methods and are much easier to implement for vehicular nodes as they have predictable motion paths. The probability of leaving helps to decide the movement of the vehicles, but during vehicle communication, most vehicles do not have the ability to leave the communication community until the next batch edge because it is difficult for them to hit the pace to leave the boundary zone after the present rekeying time. To resolve this issue, the leaving ratio [1,4] is introduced, which is given by

(2) $$\;\;\;\;Y=min\left(1,\frac{T_{1}}{T_{2}}\right)$$

where $T_{2}$ is the cost of time of the vehicles to leave or exit and $T_{1}$ is the batch of intervals. To understand the working of the algorithm, we have to take the following considerations.

- Consider that the vehicle can only exit when it is at a normal speed *X* then,

(3) $$X=\frac{2Y}{T_{1}}-X_{c}$$

- Consider that the vehicle spends some time to reach a speed *S*, then,

(4) $$S=X_{c}+a.T_{1}+\frac{1}{2}\left(2a+\left(X_{c}.T_{1}-Y\right)\right)+a^{2}.T_{1}^{2}$$

The structure of the network and the basic key handover algorithm is inherited from the blockchain-based key inheritance scheme. To have a fair metric for calculating the output, a time interval of one second is used as the typical metric for measuring the efficiency of various selection times.

## 5. Results

The longer collection cycle here, under moderate traffic constraints, provides less main handover operation time. A rapid collection duration, however, and shorter collection cycles work best under high traffic pressure. This ensures that the duration of the collection cycle should be changed in terms of traffic in order to reduce the main handover transfer time.

The main manager is the certificate authority who manages many RSUs to provide a wide area of network coverage. The vehicle contact network messages require autonomous control by service manager cells since the Vehicle contact is meant to be used on a broad scale of the regional territory. The service managers carry on much of the role of message delivery, and a single service manager manages multiple Road Side Units (RSUs). Thus, the service manager serves as the main key manager in this case, as well as a conduit between the vehicle nodes and the controller department. In crowded traffic conditions, the number of cars and rows is considered. In the worst situation, the polluted traffic is directed at analyzing our scheme (as well as the worst strain of vehicular communication). In this example, higher speed levels are not included since they reflect the off-peak traffic conditions (not the worst case) with fewer participant nodes [40]. The main tree structure of this scenario is based on the Logical Key Hierarchy (LKH) [41,42] to boost the rekeying performance. LKH is very useful when handling different issues related to keying in secure communication between the groups. It helps in reducing the storage requirements and minimizing the number of transmissions [36]. This is very useful in autonomous vehicles in which multiple instructions are transmitted in a short time to avoid any confusion.

For binary tree degrees as low tree degrees, further individual node encryption results after rekeying. The cryptographic systems in the scheme have been verified for use as an AES-CCM asymmetry encryption scheme as a symmetry scheme and the production of digital signatures using the Elliptic Curve Digital Signature Algorithm (ECDSA). Elliptic-curve cryptography (ECC) with elliptic curve secp160r1 in Crypto++ [42] is not only chosen for the ECIES cryptographic scheme but also for the ECDSA digital signature scheme. The Elliptic Curve Integrated Encryption Scheme (ECIES) ciphertext length has 75 bytes, which provides a stronger degree of reliability. For the Advanced Encryption Standard Counter Mode with Cipher Block Chaining Message Authentication Code (AES-CCM) scheme, the group key (GK) is the hidden key. The message payload ciphertext has 32 bits, which helps achieve better processing efficiency and a reasonable level of protection. All the physical ECDSA signatures have a total of 42 bits. Since core procedures in the Vehicular Communication Systems (VCSs) are simpler than those transitions between conventional wireless-mobile-specific transaction amounts. The key initialization structure of the system is recorded in Table 2.

**Figure 3:**
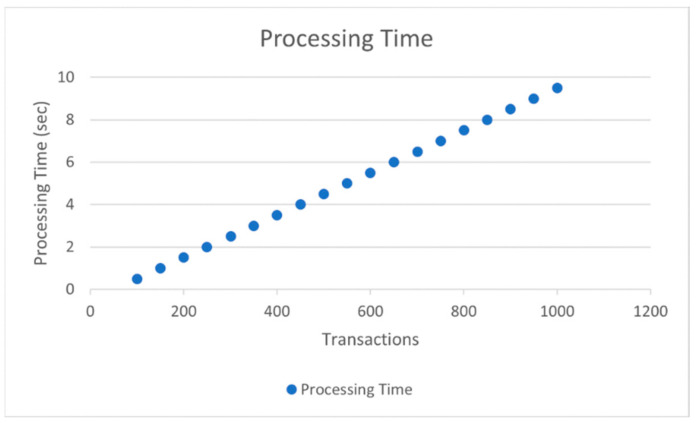
Block preparation timings with regards to the transaction number.

**Figure 4:**
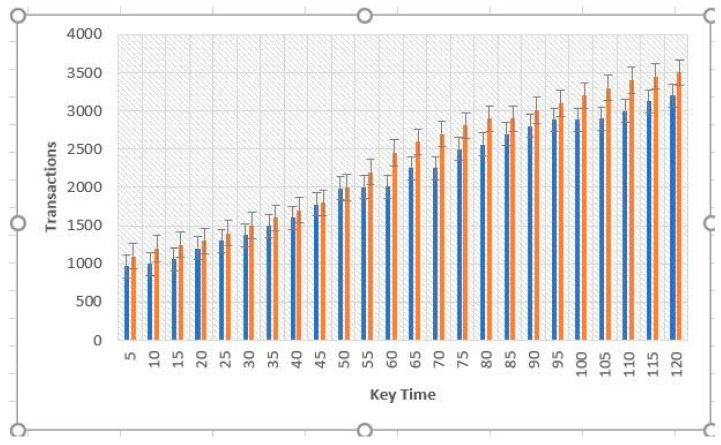
Comparison of time cost values within the same networks. The orange bars show hashgraph transactions, and the blue bars show the blockchain transactions.

Upon analysis, we have found that the planning time increases exponentially regarding transaction number production. Before 350 transactions, the transfer time gradually increases quasi-linearly. The processing time is over 0.25 s if the transaction is greater than 450. Finally, as there are 15,000 orders, the planning time hits 1.05 s. The non-linear graph initially grows exponentially at initial rates for some time but then becomes linear later Figure 3 and Figure 4.

Key-transmission efficiency is calculated by block propagation time from the present manager of operation to the manager of the destination service, as shown in Table 2, Table 3 and Table 4.

A lower transaction processing period will likely allow the service manager to introduce further cross-border requests into the blocks. Likewise, a larger number of neighbors lengthens the primary transmitting time. All of these forms are similar to adding an amount of flow. For clarification, we find the volume of traffic below 4000 vehicles an hour to be the off-peak mode, while the number of vehicles above 14,000 an hour is known as the peak mode. The blockchain framework takes more time to process as there are 2000 vehicles running per hour on the lane. That is because preparing and processing blocks take more time than just the authentication and encryption of one or two letters. However, as can be seen from the results of 7000 vehicles per hour, time costs in the conventional system increase proportionally to the volume of traffic, although the results in the blockchain system stay constant with respect to various volumes of traffic. In fact, the time loss of conventional layout approaches the blockchain outcomes as adjacent cells and exceeds 2, and transaction aggregation approaches exceed 0.3 s. The response time of the conventional layout decreases dramatically as traffic volumes rise. In principle, when the conventional network handles messages one by one, the time cost of transmission will rise in proportion to the transactions. This is shown in Figure 3 and Figure 4. Time costs under a 2.0 s transaction collection time double the amount under 1.0 s collection time because double the collection time means that there are nearly two cycles more of cross-border demands. Similarly, six neighbors’ findings are nearly double the time expense of three neighbors. The time factor in the blockchain network, on the other hand, reveals a smooth and stable surface under any traffic conditions since transfers are aggregated into a single node of the graph and analyzed one node each time, no matter how many network transactions.

## 6. Conclusions

In this paper, we used an IoT roadmap for the private information of individuals inside the IoT ecosystem such that security is prevented from malicious users. In fact, privacy security protects an IoT, and the results showed that the cost of the transmission of keys is efficient, secure and reliable in the hashgraph network compared to traditional methods. The findings show that the crucial turnaround time exponentially rises according to the rise in traffic volumes. The future Internet of Things (IoT) roadmap suggests everything, including the specifics of human life, will be linked. For this reason, the private information of individuals inside the IoT ecosystem is compromised by malicious users. In fact, privacy security protects an IoT system from concerted attacks, as adversaries are unable to concentrate their attack on a single unit. To resolve the privacy issue, future work will concentrate on further examining privacy concerns, including exploring a framework that offers protection as well as safety. The future research will be established as a plan to complement existing commitments.

## Figures and Tables

**Table 1:** Existing threats to VANTEs and their effects [35,36,37,38,39].

Existing Threats to VANETs	Effect on VANETs
Unauthorized access of data	The VANET suffers from hacking and data modification
Denial of Service (DoS) and DDoS	The VANET suffers from malicious actions affecting the reliability of the system
Challenges relates to data transfer	The VANET suffers from data loss and unauthorized data access
IoT Security	The VANET suffers from network hacking
Storage and Sharing and information	Insufficient storage for network information

**Table 2:** Key Initialization.

Steps	Timestamp
Joining of Vehicles	0 ms
Registration of vehicles	3.21200 ms
Service manager receives the messages	4.01456 ms
Service manager verifies the messages	6.23542 ms
The message is received by PKI	7.02354 ms
The message verified by PKI	7.11203 ms
Preparation of rekeying messages by service manager	7.4102 ms
Total preparation time of rekeying messages	5.2895 ms
Messages sent	t_send_
Messages received by the vehicles	t_send_ + 0.18765 ms

**Table 3:** Key transferring time for a traffic level of 3000 to 9000 vehicles/road/h (Traffic Level/T = Transaction Collection Time).

T	3000	4000	5000	6000	7000	8000	9000
0.5 s	0.200	0.187	0.223	0.198	0.215	0.222	0.211
1.0 s	0.281	0.256	0.233	0.248	0.258	0.325	0.336
1.5 s	0.235	0.227	0.310	0.311	0.346	0.398	0.417
2.0 s	0.245	0.265	0.279	0.387	0.411	0.454	0.422
2.5 s	0.201	0.288	0.294	0.331	0.337	0.340	0.440
3.0 s	0.230	0.290	0.301	0.311	0.333	0.425	0.478
3.5 s	0.231	0.228	0.296	0.324	0.328	0.371	0.388

**Table 4:** Parameters estimated during a probability-based scenario.

RSU Coverage Areas	1000 m
Message Transmission Power	30 Mw
Amount of Vehicles	220 Vehicles
Number of Roads	14 rows
Rekeying Intervals	1 s
Standard deviation of traffic distribution function (TDF)	7.02
Mean of TDF	50.25
Degree of Key Tree	binary

## Data Availability

Not applicable.

## References

[1] Lei A., Cruickshank H., Cao Y., Asuquo P., Ogah C.P.A., Sun Z. (2017). Blockchain-Based Dynamic Key Management for Heterogeneous Intelligent Transportation Systems. IEEE Internet Things J..

[2] Soto Villacampa J.A. (2019). Towards a Blockchain-Based Private Road Traffic Management Implementation. Ph.D. Thesis.

[3] (2015). Self-Driving Cars Could Reduce Accidents by 90%, Saving $190B—Goliath. https://www.goliath.com/tech/self-driving-cars-could-reduce-accidents-by-90-saving-190b/.

[4] Lei A. (2017). Dynamic Key Management for Secure Heterogeneous Vehicular Communication Systems.

[5] Sharma R., Chakraborty S. Blockapp: Using blockchain for authentication and privacy preservation in IoV. Proceedings of the 2018 IEEE Globecom Workshops (GC Wkshps).

[6] Nakamoto S. Bitcoin: A Peer-to-Peer Electronic Cash System. bitcoin.org.

[7] Devi E.A., Ramya K.C., Kumar K.S., Ahmad S., Kadry S., Park H.J., Kang B.G. (2022). Energy Aware Metaheuristic Optimization with Location Aided Routing Protocol for MANET. CMC-Comput. Mater. Contin..

[8] Barskar R., Ahirwar M., Vishwakarma R. Secure key management in vehicular ad-hoc network: A review. Proceedings of the 2016 International Conference on Signal Processing, Communication, Power and Embedded System (SCOPES).

[9] Sasikala G., Dhanalakshmi K.S. (2012). Key management techniques for vanets. Intl. J. Comput. Appl..

[10] Tan H., Chung I. (2019). Secure Authentication and Key Management with Blockchain in VANETs. IEEE Access.

[11] Shrestha R., Bajracharya R., Shrestha A.P., Nam S.Y. (2020). A new type of blockchain for secure message exchange in VANET. Digit. Commun. Netw..

[12] Ma Z., Zhang J., Guo Y., Liu Y., Liu X., He W. (2020). An Efficient Decentralized Key Management Mechanism for VANET With Blockchain. IEEE Trans. Veh. Technol..

[13] Li B., Liang R., Zhu D., Chen W., Lin Q. (2021). Blockchain-Based Trust Management Model for Location Privacy Preserving in VANET. IEEE Trans. Intell. Transp. Syst..

[14] (2020). 5 Key Challenges For Blockchain Adoption In 2020|Blockchain Council. https://www.blockchain-council.org/blockchain/5-key-challenges-for-blockchain-adoption-in-2020/.

[15] Baird L., Harmon M., Madsen P. (2019). A Public Hashgraph Network & Governing Council.

[16] (2018). Hedera Hashgraph Problems and Challenges of Implementation. https://applicature.com/blog/blockchain-technology/hashgraph-problems.

[17] Schmoll A. (2018). A Hardware-Based Secure Communication Module to Protect Internet Connected Vehicles. Master’s Thesis.

[18] Rui S. (2018). Design and Performance Analysis of Urban Traffic Control Systems. https://ethos.bl.uk/OrderDetails.do?uin=uk.bl.ethos.747226.

[19] Alshamrani S.S., Jha N., Prashar D. (2021). B5G Ultrareliable Low Latency Networks for Efficient Secure Autonomous and Smart Internet of Vehicles. Math. Probl. Eng..

[20] Lasla N., Younis M., Znaidi W., Ben Arbia D. Efficient distributed admission and revocation using blockchain for cooperative its. Proceedings of the 2018 9th IFIP International Conference on New Technologies, Mobility and Security (NTMS).

[21] Lu Z., Wang Q., Qu G., Liu Z. BARS: A Blockchain-Based Anonymous Reputation System for Trust Management in VANETs. Proceedings of the 2018 17th IEEE International Conference on Trust, Security and Privacy in Computing and Communications/12th IEEE International Conference on Big Data Science and Engineering (TrustCom/BigDataSE).

[22] Yang Y.-T., Chou L.-D., Tseng C.-W., Tseng F.-H., Liu C.-C. (2019). Blockchain-Based Traffic Event Validation and Trust Verification for VANETs. IEEE Access.

[23] Leiding B., Vorobev W.V. (2018). Tezos-Based Vehicular Ad Hoc Blockchains. https://uploads-ssl.webflow.com/5a4ea18a81f55a00010bdf45/5b9599af6708aa28b5e2a7b5_20180908_draft-v05.pdf.

[24] Prashar D., Jha N., Jha S., Joshi G., Seo C. (2020). Integrating IoT and Blockchain for Ensuring Road Safety: An Unconventional Approach. Sensors.

[25] Sharma S., Agarwal V., Gupta D., Hasija V. Directed Acyclic Graph for Peer-to-Peer Distributed Communication in Vehicular Network. https://githubmemory.com/repo/rubiruchi/Directed-Acyclic-Graph-for-Peer-to-Peer-Distributed-Communication-in-Vehicular-Network.

[26] Wang G., Wang B., Wang T., Nika A., Zheng H., Zhao B.Y. Defending against Sybil devices in crowdsourced mapping services. Proceedings of the 14th Annual International Conference on Mobile Systems Applications, and Services.

[27] Liu X., Huang H., Xiao F., Ma Z. (2020). A Blockchain-Based Trust Management with Conditional Privacy-Preserving Announcement Scheme for VANETs. IEEE Internet Things J..

[28] Javaid U., Aman M.N., Sikdar B. DrivMan: Driving Trust Management and Data Sharing in VANETs with Blockchain and Smart Contracts. Proceedings of the 2019 IEEE 89th Vehicular Technology Conference (VTC2019-Spring).

[29] Oham C., Michelin R.A., Jurdak R., Kanhere S.S., Jha S. (2021). B-FERL: Blockchain based framework for securing smart vehicles. Inf. Process. Manag..

[30] Bagga P., Sutrala A.K., Das A.K., Vijayakumar P. (2021). Blockchain-based batch authentication protocol for Internet of Vehicles. J. Syst. Arch..

[31] Javaid U., Aman M.N., Sikdar B. (2020). A Scalable Protocol for Driving Trust Management in Internet of Vehicles with Blockchain. IEEE Internet Things J..

[32] Xu Z., Liang W., Li K.-C., Xu J., Jin H. (2021). A blockchain-based Roadside Unit-assisted authentication and key agreement protocol for Internet of Vehicles. J. Parallel Distrib. Comput..

[33] (2021). Hashgraph Vs Blockchain: A Detailed Comparison. https://101blockchains.com/hashgraph-vs-blockchain/.

[34] Abu Ali N., Taha A.-E.M., Barka E. (2020). Integrating Blockchain and IoT/ITS for Safer Roads. IEEE Netw..

[35] Tripathi G., Ahad M.A., Sathiyanarayanan M. The Role of Blockchain in Internet of Vehicles (IoV): Issues, Challenges and Opportunities. Proceedings of the 2019 International Conference on contemporary Computing and Informatics (IC3I).

[36] (2022). How Blockchain Will Revolutionize Future Cars. https://www.investopedia.com/investing/how-blockchain-will-revolutionize-future-cars/.

[37] (2018). Hedera Hashgraph vs. Blockchain|Comparison. https://www.leewayhertz.com/hashgraph-vs-blockchain/.

[38] Shrestha R., Bajracharya R., Nam S.Y. (2018). Challenges of Future VANET and Cloud-Based Approaches. Wirel. Commun. Mob. Comput..

[39] Toulni H., Boudhane M., Nsiri B., Miyara M. (2016). An Adaptive Key Exchange Procedure for VANET. Int. J. Adv. Comput. Sci. Appl..

[40] Wong C.K., Gouda M., Lam S. (2000). Secure group communications using key graphs. IEEE/ACM Trans. Netw..

[41] Harney H., Harder E. (1999). Logical Key Hierarchy Protocol. https://datatracker.ietf.org/doc/html/draft-harney-sparta-lkhp-sec-00.txt.

[42] Pande A.S., Thool R.C. Survey on Logical Key Hierarchy for secure group communication. Proceedings of the 2016 International Conference on Automatic Control and Dynamic Optimization Techniques (ICACDOT).

